# Effect of anti-inflammatory therapy on vascular biomarkers for subclinical cardiovascular disease in rheumatoid arthritis patients

**DOI:** 10.1007/s00296-022-05226-w

**Published:** 2022-10-21

**Authors:** Annelies B. Blanken, Reinder Raadsen, Rabia Agca, Alper M. van Sijl, Yvo M. Smulders, Michael T. Nurmohamed

**Affiliations:** 1 Amsterdam Rheumatology and Immunology Center, Location Reade, Department of Rheumatology, PO box 58271, 1040 HG Amsterdam, the Netherlands; 2 Amsterdam Rheumatology and Immunology Center, Location Amsterdam UMC, Department of Rheumatology, VU University Medical Center, Amsterdam, The Netherlands; 3 Amsterdam UMC, Department of Internal Medicine, VU University Medical Center, Amsterdam, The Netherlands

**Keywords:** Rheumatoid arthritis, Atherosclerosis, Intima-media thickness, Pulse wave velocity, Arterial stiffness, Anti-inflammatory therapy

## Abstract

**Objective:**

To assess the effect of 4 years of anti-inflammatory therapy on markers of subclinical vascular disease in rheumatoid arthritis patients.

**Methods:**

Carotid intima media thickness (IMT), augmentation index (AIx@75) and pulse wave velocity (PWV) measurements were performed repeatedly in 61 RA patients (30 early RA starting with csDMARDs and 31 established RA starting with adalimumab) for 4 years. These markers were also measured in 29 controls with osteoarthritis at baseline (BL).

**Results:**

IMT and AIx@75 at BL were higher in RA compared to OA, while PWV was higher in OA. In RA patients, AIx@75 and PWV decreased in the first 6 months after starting anti-inflammatory therapy. At 48 M, the level of AIx@75 remained lower than before therapy, while PWV at 48 M was comparable to BL (AIx@75: BL 28% (95% confidence interval 25–30%), 6 M 23% (20–26%), 48 M 25% (22–28%); PWV: BL 8.5 (7.8–9.2), 6 M 8.0 (7.1–8.9), 48 M 8.6 (7.6–9.6) m/s). IMT remained stable. There was an effect of disease activity (longitudinally, adjusted for changes over time) on IMT, AIx@75 and PWV.

**Conclusion:**

This study suggests modest beneficial changes in some surrogate markers of subclinical vascular disease after anti-inflammatory therapy. These changes were associated with improvement in disease activity markers. Whether or not these beneficial changes ultimately predict a reduction in clinicalcardiovascular endpoints remains to be established in prospective studies.

**Supplementary Information:**

The online version contains supplementary material available at 10.1007/s00296-022-05226-w.

## Introduction

Rheumatoid arthritis (RA) is a chronic disease characterized by systemic inflammation, mainly affecting the joints. RA is associated with an increased risk of cardiovascular disease (CVD) and CVD-associated mortality [1–3]. Traditional cardiovascular (CV) risk factors in RA only partly explain the increased CVD risk in these patients. This suggests that chronic systemic inflammation itself contributes to (accelerated) atherosclerotic plaque development and/or instability [1, 4, 5]. Strategies to reduce CV risk in RA are modification of traditional risk factors (e.g. lifestyle recommendations, antihypertensive treatment, and cholesterol lowering agents), but accumulating evidence suggest that anti-inflammatory treatment (e.g. with conventional synthetic disease modifying anti-rheumatic drugs (csDMARDs) or biological DMARDs (bDMARD, such as TNF-blockers) also reduces CV risk in RA patients [6–9].

Established markers for subclinical vascular disease are carotid intima media thickness (IMT; thickness of inner layer of the arterial wall), pulse wave velocity (PWV; represents arterial wall stiffness) and augmentation index (AIx; another measure for arterial stiffness) [10–13]. These markers are often used in trials to assess the effect of interventions on CV risk [14]. Studies to assess whether short-term anti-inflammatory therapy (follow-up period ranging between 1 and 12 months) has a favorable effect on these surrogate markers are inconclusive [15–18]. This is the first study to investigate the long-term effect of anti-inflammatory.

The aim of this explorative study was to assess the effect of 4 years of anti-inflammatory therapy on IMT, AIx and PWV in RA patients. Insight in these surrogate markers and the changes over time after anti-inflammatory treatment can provide additional information on the treatment of cardiovascular comorbidities in RA.

## Methods

### Study participants

Participants with active RA (i.e. disease activity score of 28 joints with erythrocyte sedimentation rate (DAS28-ESR) ≥ 4) of ≥ 50 years starting with anti-inflammatory treatment, and age-and sex-matched osteoarthritis (OA) controls, were recruited between October 2011 and February 2017 from outpatient clinics of the departments of Rheumatology of Reade and Amsterdam UMC, location VUmc, Amsterdam, the Netherlands, as described previously [19, 20]. Exclusion criteria were active tuberculosis or other severe infections, pregnancy, moderate to severe heart failure (NYHA class III/IV), cancer, and life expectancy < 12 months. Patients were categorized into three groups: (1) newly diagnosed patients with early RA, not yet on DMARD therapy, and starting with methotrexate (MTX) or another csDMARD (sulfasalazine or hydroxychloroquine, whether or not combined with short-term glucocorticoids), (2) patients with established RA, already on csDMARDs, and starting with a first biological DMARD (adalimumab) in combination to the csDMARDs they already used prior to the study and (3) age- and sex-matched controls with OA of the knee or hip, not using DMARD of corticosteroid therapy. The study was approved by the Slotervaartziekenhuis and Reade medical ethics committee (protocol number: NL34047.048.10, approval date: 23–12-2010) and a written informed consent form was obtained from all participants, according to the principles of the Declaration of Helsinki.

### Study visits

For RA patients, study visits were scheduled at baseline (BL) and 6, 12, 24, 36 and 48 months after starting treatment. Patients who discontinued treatment of interest (csDMARDs for early RA and adalimumab for established RA) during follow-up were excluded as per protocol, but they were invited for the 6- and 48-month visits to be included in the intention-to-treat analysis. Therefore, intention-to-treat analysis only includes the visits at BL, 6 and 48 months. OA controls were measured once, in order to cross-sectionally compare baseline measurements in RA with a group of patients that also suffers from joint problems and subsequent mobility limitations (resulting in lower physical activity), but without systemic inflammation.

### Surrogate markers

Surrogate markers (IMT, AIx@75 and PWV) were measured by three observers (ABB, RA, AMvS), all trained by the same trainers of the Clinical Research Unit, Internal Medicine, Amsterdam UMC, location VUmc. Each observer performed a reproducibility test with the trainer before starting to perform measurements (a maximum of 10% inter- and intra-observer variability was allowed).

Mean carotid IMT of three measurements 10 mm proximal to the carotid bifurcation in the right common carotid artery (far wall) was acquired using Artlab echotracking system with a 7.5 MHz linear probe (Esaote, Maastricht, the Netherlands), according to Mannheim consensus [21]. Pulse wave analysis was performed with applanation tonometry (SphygmoCor, AtCor medical, Sydney, Australia). Carotid and femoral pulse waves were measured sequentially and gated by a simultaneously recorded ECG signal. Carotid-femoral PWV was determined by the transit time of the pulse wave between the carotid and femoral artery divided by the distance between the two recording sites (m/s). For AIx, the central aortic waveform was mathematically derived from the radial pulse wave. AIx was then determined by subtracting the first from the second (the augmented) systolic pressure peak of the central aortic waveform and expressed as a percentage of the pulse pressure. Mean of three measurements was taken and normalized to heart rate of 75 beats/minute (AIx@75).

### Clinical assessments

Demographic data, medical history, medication use, smoking status, erythrocyte sedimentation rate (ESR), C-reactive protein (CRP), DAS28-CRP, health assessment questionnaire (HAQ), blood pressure (BP), heart rate, hypertension (systolic BP > 140 mmHg and/or diastolic BP > 90 mmHg and/or currently on antihypertensive treatment), body mass index (BMI), total cholesterol (TC), high-density lipoprotein cholesterol (HDLc), low-density lipoprotein cholesterol (LDLc) and triglycerides (TG) were assessed at all visits. Good response to therapy was defined as achieving low disease activity on DAS28-CRP after 6 months, with low disease activity defined as DAS28-CRP < 2.7, according to [22]).

### Statistical analysis

Data are presented as mean ± standard deviation (SD) for normally distributed variables, median with interquartile range (IQR) for skewed continuous variables, or frequencies for categorical variables. Normality of dependent variables, or residuals after regression analysis, were assessed by visual inspection of the histogram and quantile–quantile plot. For regression analyses, linearity and homoscedasticity were assessed by plotting residuals versus predicted values and observed versus predicted values. Cross-sectional differences in surrogate markers between the groups were assessed with independent *t* test. Longitudinal analysis was done with linear mixed-effect models, allowing adjustment for the potential correlations between repeated measurements within the same patients over time. A random intercept was added to all models, while log-rank test was done to determine whether a random slope (with unstructured correlation matrix) was necessary. Because of slight heteroscedasticity (mainly for PWV), robust standard errors were estimated. For the analysis comparing response versus non-response, a visit was marked as good response if the patient had DAS28-CRP < 2.7 during that visit. To analyze the effect of four estimates of disease activity (ESR, CRP, DAS28-CRP and HAQ) on the surrogate markers, adjusting for the changes over time, we used univariate linear mixed effect models with time added as covariate to the model. For this analysis, standardized regression coefficients are reported, representing the number of SDs that the surrogate markers change, when the independent variable changes one SD. Data analysis was performed using SPSS version 24 and STATA SE 13.

## Results

The study population consisted of 61 RA patients (30 early RA and 31 established RA patients) and 29 age-and-sex-matched controls with OA. Baseline characteristics are shown in Table 1. During the 48 months follow-up, 19 (32%) patients dropped out (*n* = 6, 5, 2, 3 and 3 at month 6, 12, 24, 36 and 48, respectively). Reasons were withdrawal of informed consent (*n* = 6), lost-to-follow-up (*n* = 7) and death (*n* = 6). In total, 227 visits were performed, and IMT, AIx@75 and PWV were measured in 92%, 84% and 73% of the visits, respectively. Main reasons for not having a measurement were material breakage, unavailability of equipment due to maintenance and measurement failure due to irregular heartbeat or weak signal because of cervical adipose tissue. The number of patients per visit is shown in Supplementary Fig. 1.

**Table 1:** Baseline characteristics

	Early RA		Established RA		OA	
	*n* = 30		*n* = 31		*n* = 29	
*Demographics*						
Age (years), mean ± SD	64	± 9	60	± 7	62	± 6
Female, n (%)	17	(57%)	17	(55%)	16	(55%)
*Rheumatic disease*						
Disease duration (years), median (IQR)	0.04	(0.02–0.06)	8	(2–14)	8	(2–11)
IgM-RF and/or ACPA positive, n (%)	21	(70%)	22	(71%)	n/a	
DAS28-CRP, mean ± SD	4.4	± 1.0	4.1	± 1.0	n/a	
ESR (mm/h), median (IQR)	25	(14–29)	18	(7–26)	7	(5–14)
CRP (mg/L), median (IQR)	10	(1–30)	4	(1–16)	1	(1–3)
Methotrexate, *n* (%)	0	(0%)	24	(77%)	n/a	
Other csDMARD, *n* (%)	0	(0%)	17	(55%)	n/a	
bDMARD, *n* (%)	0	(0%)	0	(0%)	n/a	
Corticosteroids, *n* (%)	1	(3%)	13	(42%)	n/a	
*Cardiovascular risk factors*						
Current smoking, *n* (%)	6	(20%)	8	(26%)	1	(3%)
Pack years, median (IQR)	6	(0–23)	10	(0–30)	0.5	(0–13)
Diabetes mellites, *n* (%)	5	(17%)	5	(16%)	3	(10%)
Body mass index (kg/m^2^), mean ± SD	27	± 5	28	± 7	29	± 5
History of CVD, *n* (%)	5	(17%)	9	(29%)	9	(31%)
Hypertension, *n* (%)	17	(57%)	18	(58%)	19	(66%)
Systolic BP (mmHg), mean ± SD	142	± 21	127	± 17	133	± 19
Diastolic BP (mmHg), mean ± SD	85	± 8	78	± 10	81	± 9
TC/HDLc ratio, mean ± SD	4.0	± 1.6	3.0	± 0.9	3.5	± 1.1
TC (mmol/L), mean ± SD	4.8	± 1.8	4.9	± 1.0	4.9	± 1.0
HDLc (mmol/L), mean ± SD	1.3	± 0.3	1.8	± 0.8	1.5	± 0.4
LDLc, (mmol/L), mean ± SD	3.0	± 1.0	2.7	± 0.7	2.8	± 0.9
Triglycerides (mmol/L), median (IQR)	1.0	(0.9–1.6)	0.9	(0.8–1.3)	1.1	(0.9–1.6)
Antihypertensive drug, *n* (%)	16	(53%)	14	(45%)	17	(59%)
Statin, n (%)	6	(20%)	9	(29%)	12	(41%)
Anticoagulants, *n* (%)	7	(23%)	8	(26%)	12	(41%)
Vascular biomarkers (study outcomes)*						
IMT (mm), mean ± SD	0.68	± 0.10	0.66	± 0.12	0.64	± 0.09
AIx@75 (%), mean ± SD	29	± 10	27	± 11	24	± 10
PWV (m/s), mean ± SD	8.8	± 2.3	8.2	± 2.8	9.9	± 2.3

*Statistical comparisons of the baseline values of the study outcomes are presented in the text. ACPA: anti-citrullinated protein antibodies, AIx@75: augmentation index normalized to heart rate of 75 beats/minute, bDMARD: biological disease modifying anti-rheumatic drug, BP: blood pressure, CRP: C-reactive protein, CVD: cardiovascular disease, csDMARD: conventional synthetic disease-modifying anti-rheumatic drugs, DAS28: disease activity score of 28 joints, ESR: erythrocyte sedimentation rate, HDLc: high-density lipoprotein cholesterol, IgM-RF: rheumatoid factor, IMT: intima media thickness, IQR: interquartile range, LDLc: low-density lipoprotein cholesterol, OA: osteoarthritis, PWV: pulse wave velocity, RA: rheumatoid arthritis, SD: standard deviation, TC: total cholesterol

As shown in Table 1, 14 (23%) RA patients had a history of CVD at baseline, including cerebrovascular event (*n* = 6), acute coronary syndrome (*n* = 5), congestive heart failure (*n* = 1), peripheral artery disease (*n* = 1) and acute coronary syndrome plus congestive heart failure (*n* = 1). During follow-up, 2 patients developed CVD (fatal cerebrovascular event (*n* = 1) and non-fatal acute coronary syndrome (*n* = 1), both in early RA group) and 5 patients deceased of non-CVD related causes.

### Cross-sectional comparison RA and OA patients

When comparing RA (all RA patients combined) with OA at baseline (Table 1), mean values of IMT and AIx@75 were slightly higher for the RA group (IMT: RA 0.67 ± 0.11 vs. OA 0.64 ± 0.09, *p* = 0.19, AIx@75: RA 28 ± 10 vs. OA 24 ± 10, *p* = 0.19). In contrast, PWV was higher in OA patients (RA 8.5 ± 2.5 vs. OA 9.9 ± 2.3, *p* = 0.053). Stratified analysis comparing early with established RA showed that all indices of subclinical vascular disease were highest for early RA patients, who also had highest RA disease activity and blood pressure (IMT: early RA 0.68 ± 0.10 vs. established RA 0.66 ± 0.12, AIx@75: early RA 29 ± 10 vs. established RA 27 ± 11, PWV: established RA 8.8 ± 2.3 vs. 8.2 ± 2.8, all statistically non-significant p > 0.05).

### Longitudinal intention-to-treat analysis

In the early RA group, 23 (85%) patients had started with csDMARD monotherapy, 2 (7%) with methotrexate + other csDMARD(s) and 1 (4%) with csDMARD(s) other than methotrexate. In the established RA group, 4 (15%) patients had started with adalimumab as monotherapy and 21 (78%) as addition to the csDMARDs they were already using prior to the study (12 (44%) methotrexate, 2 (7%) other csDMARDs and 7 (26%) methotrexate + other csDMARDs). An overview of anti-inflammatory treatment use per study visit is shown in Supplemental Fig. 1. In total, 36 (59%) of the patients achieved low disease activity at 6 months after starting treatment (defined as ‘response’).

**Fig. 1:**
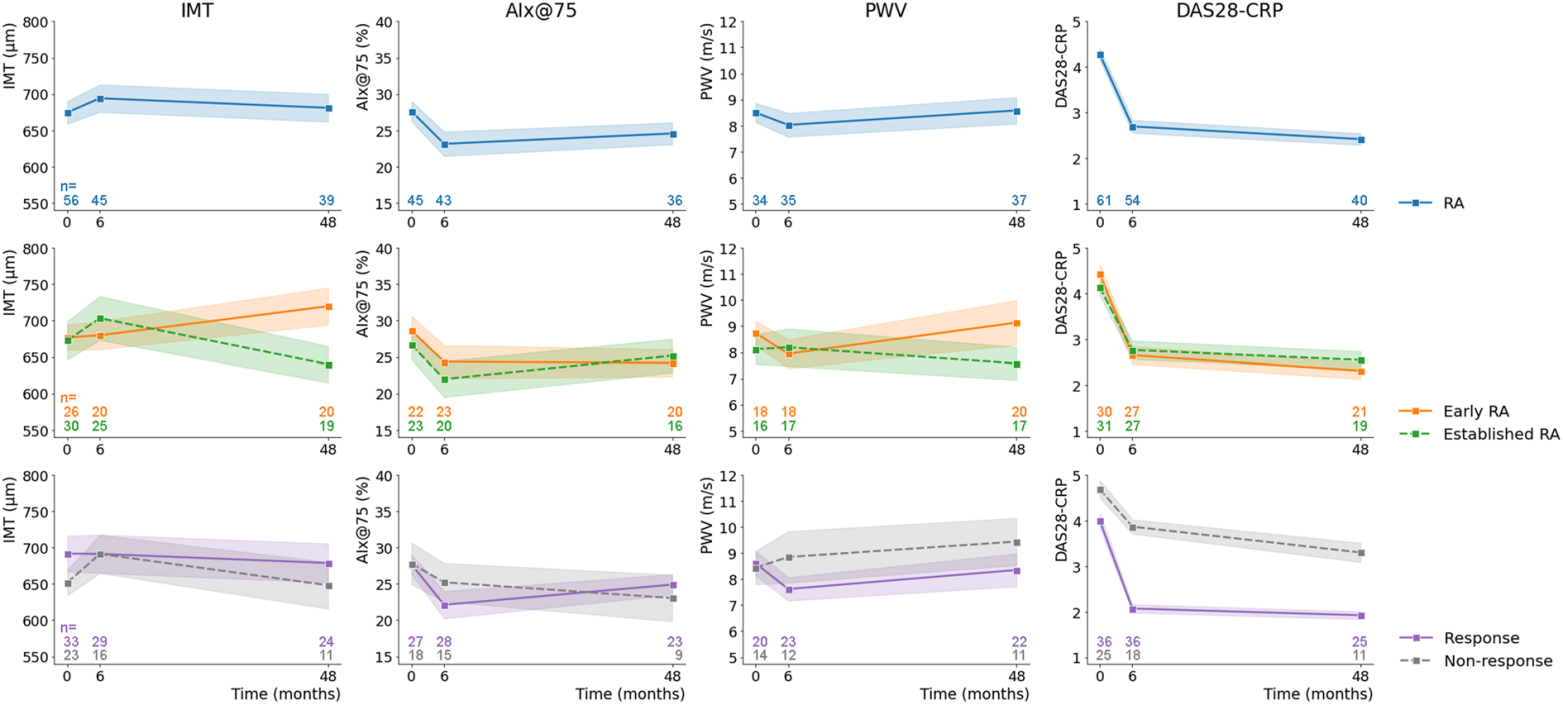
Longitudinal effect of anti-inflammatory treatment on surrogate markers and disease activity: Intention-to-treat analysis. Results of univariate linear mixed-effects models (with random intercept, and random slope when necessary (log-rank test)), with robust standard errors, AIx@75: augmentation index normalized to heart rate of 75 beats/minute, CRP: C-reactive protein, DAS28: disease activity score of 28 joints, IMT: intima media thickness, PWV: pulse wave velocity, RA: rheumatoid arthritis. Intention-to-treat analysis. For exact numbers refer to Supplemental Table 1

In the total group of RA patients, AIx@75 decreased in the first 6 months after starting anti-inflammatory therapy, while PWV showed a decreasing trend, particularly in responding patients (Fig. 1, Supplemental Table 1, *p* < 0.001 and *p* = 0.103, respectively). At 48 months, the level of AIx@75 still remained lower than before therapy (*p* = 0.016), while PWV at 48 months was comparable to that at BL (*p* = 0.900). In contrast, IMT seemed to slightly increase at 6 months (*p* = 0.257), but was comparable to BL at 48 months (*p* = 0.788).

Stratification for early and established RA showed that both IMT and PWV decreased over the 4 years for established RA, while increasing over time for early RA patients (non-significant, Supplemental Table 1). For AIx@75 there was no clear difference between the groups.

Stratification for response and non-response showed that mainly in the first 6 months there was a difference between these two groups: IMT remained stable in responders, while it increased in non-responders (*p* = 0.998 and *p* = 0.050, respectively), AIx@75 decreased slightly more for responders (*p* = 0.001 for responders, *p* = 0.150 for non-responders), and PWV decreased in responders, while increased in non-responders (*p* = 0.003 and *p* = 0.552, respectively) (Supplemental Table 1). For IMT and AIx, these minor short-term differences did not persist on long term.

Table 2 shows the effect of disease activity on IMT, AIx@75 and PWV, adjusted for time. All three surrogate markers were positively associated with serological inflammatory markers: when ESR or CRP increased with 1 SD, the surrogate markers increased with 0.09 to 0.24 SDs. In addition, DAS28-CRP and HAQ were positively associated with AI@75 and PWV (betas 0.19–0.24).

**Table 2:** The effect of disease activity measures on IMT, AIx@75 and PWV: Intention-to-treat analysis

	IMT			AIx@75			PWV		
	Beta^a^	95% CI	*p*-value	Beta^a^	95% CI	*p*-value	Beta^a^	95% CI	*p*-value
ESR^b^	0.24	(0.11–0.39)	0.001	0.17	(-0.01–0.34)	0.072	0.19	(0.01–0.38)	0.038
CRP^b^	0.14	(-0.01–0.29)	0.073	0.15	(0.01–0.29)	0.031	0.09	(-0.06–0.23)	0.23
DAS28-CRP	-0.04	(-0.18–0.10)	0.56	0.19	(0.05–0.34)	0.011	0.18	(0.03- 0.32)	0.017
HAQ	0.05	(-0.09–0.20)	0.48	0.24	(0.10–0.41)	0.002	0.17	(0.03–0.30)	0.015

^a^Standardized regression coefficients of linear mixed-effects models, univariate analyses adjusted for time (with random intercept, and random slope when necessary (log-rank test))

^b^Log transformed, AIx@75: augmentation index normalized to heart rate of 75 beats/minute, CRP: C-reactive protein, DAS28: disease activity score of 28 joints, ESR: erythrocyte sedimentation rate, HAQ: health assessment questionnaire, IMT: intima media thickness, PWV: pulse wave velocity, RA: rheumatoid arthritis

### Longitudinal per protocol analysis

In total, 24 (39%) patients were excluded for the per protocol analysis, because of discontinuing treatment of interest during the 48-month period (csDMARDs for early RA or adalimumab for established RA). Reasons for discontinuing treatment were side effects (*n* = 12), treatment failure (*n* = 8), both side effects and treatment failure (*n* = 2), tapering due to good effect (*n* = 1) and practical reasons (*n* = 1). Results in patients actively on csDMARDs (early RA) or adalimumab (established RA) therapy (per protocol analysis, Supplemental Table 2), were comparable to the results in the total group of patients (intention-to-treat analysis, as described above).

## Discussion

We performed an explorative study on surrogate markers for vascular disease in RA patients starting with anti-inflammatory treatment. To our knowledge, this is the first clinical study following patients for up to 4 years after starting with anti-inflammatory therapy. Our key findings were modest beneficial changes in vascular surrogate markers after anti-inflammatory treatment, and that these changes were associated with improvement in markers of disease activity. Altogether, the results can inform researchers planning new studies on assessing the effect of anti-inflammatory treatment on the arterial system and investigating which endpoints to use.

Previous studies investigating the effect of TNF inhibiting therapy on IMT, AIx and PWV have shown conflicting results [15–18]. A systematic review concluded that there is no strong evidence for an effect of TNF inhibiting therapy on IMT, AIx and PWV [17], whereas a meta-analysis concluded that the balance of evidence suggests a beneficial effect of TNF inhibiting therapy on AIx and PWV [18]. Results of studies concerning the effect of csDMARDs are also conflicting [23–27].

In our study, we observed a short-term effect of anti-inflammatory therapy on AIx@75 and PWV at 6 months, after which both increased again. AIx@75 was still lower at 48 months than at baseline, and PWV was at a comparable level as baseline. IMT seemed to remain rather stable over the 48 months. From previous research, it was expected that without an intervention these surrogate markers would increase over time due to aging [28, 29]. As the largest reduction in inflammation is achieved within the first 6 months, it is possible that a steady-state occurs after which no further decline can be expected, and subsequent increase in the surrogate markers is due to aging. However, we did not longitudinally measure the surrogate markers in our control group, and future research with additional longitudinal measurements in a control group is warranted to investigate whether there is a delay of deterioration in the vasculature as a result of the effect by anti-inflammatory therapy. In addition, the beneficial changes we found after anti-inflammatory therapy were modest, and it can be argued whether these are clinically relevant or not. This remains to be established in future prospective studies assessing the effect of anti-inflammatory therapy on long-term CVD risk in RA patients, which could also include additional measurements for arterial calcification such as multi-slice computed tomography, and other measures relevant in the context of arterial calcification, such as lipoprotein (a) and vitamin D levels.

A large proportion of the patients in the established RA group used glucocorticosteroids at baseline. While chronic treatment with prednisone increases the risk of atherosclerotic CVD, its effects on these markers for subclinical CVD are still unclear [30, 31].

Multiple measures for RA disease activity (serological inflammatory markers, composite measure for disease activity and questionnaire for functional disability status) were associated with IMT, AIx@75 and PWV, providing further support for a link between the reduction of systemic inflammation and arterial stiffness and arterial wall thickening.

For the cross-sectional comparison we included OA patients, as these also suffer from joint problems and subsequent mobility issues, but without auto-inflammatory characteristics. The included OA patients (matched on age and sex) had highest prevalence of previous CVD and hypertension. This might be responsible for the higher PWV in OA compared to RA.

Limitations of this study are a low sample size and rather high drop-out rate, increasing the risk of selection bias. In addition, measurements were performed by different investigators at different consecutive timepoints and we therefore cannot rule out a systematic measurement difference between timepoints. Nonetheless, all three investigators were tested for inter-rater reliability by the same trainer to minimize this observer bias.

In conclusion, this study has shown modest beneficial changes in some surrogate markers of subclinical vascular disease after anti-inflammatory therapy and these changes were associated with improvement in disease activity markers. Whether or not these beneficial changes ultimately lead to a significant reduction of clinically overt cardiovascular endpoints remains to be established in prospective studies.

## Supplementary Information

Below is the link to the electronic supplementary material.

Supplementary file1 (PNG 62 KB)

Supplementary file2 (PDF 152 KB)

Supplementary file3 (PDF 92 KB)
