# Supplementary figures and images for: Effect of anti-inflammatory therapy on vascular biomarkers for subclinical cardiovascular disease in rheumatoid arthritis patients
Source: Rheumatol Int. 2022 Oct 21;43(2):315–22. doi: 10.1007/s00296-022-05226-w (PMC9898416; doi:10.1007/s00296-022-05226-w)

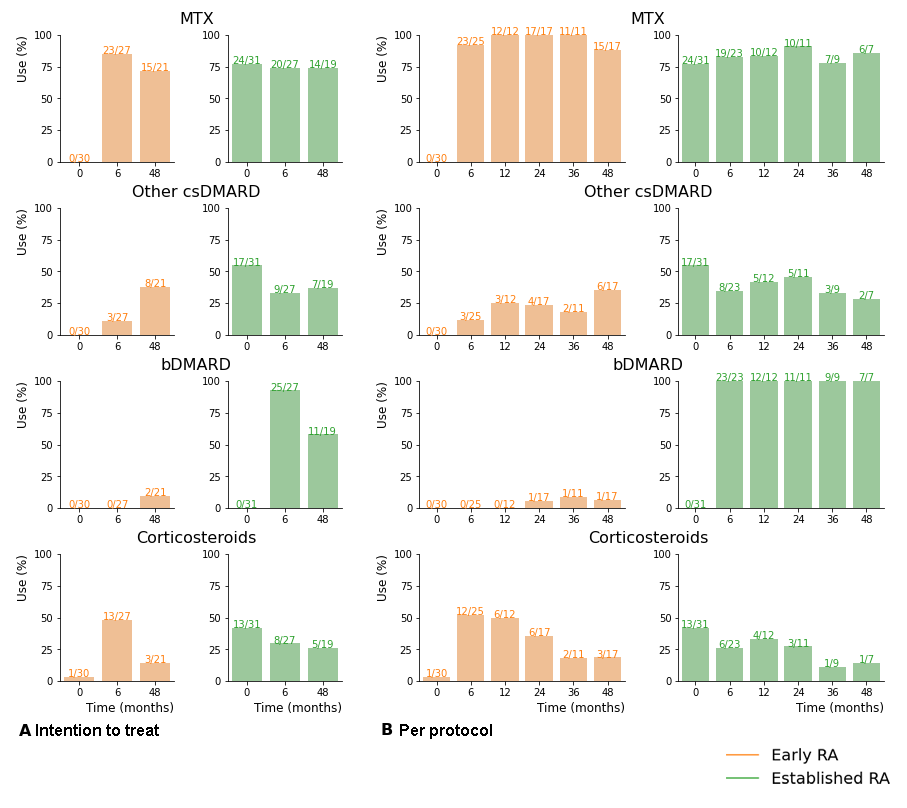

Supplement: Supplementary file 1 — Supplementary file1 (PNG 62 KB) [file 296_2022_5226_MOESM1_ESM.png]
