# Supplementary material for: Effect of anti-inflammatory therapy on vascular biomarkers for subclinical cardiovascular disease in rheumatoid arthritis patients
Source: Rheumatol Int. 2022 Oct 21;43(2):315–22. doi: 10.1007/s00296-022-05226-w (PMC9898416; doi:10.1007/s00296-022-05226-w)
Supplement: Supplementary file 2 — Supplementary file2 (PDF 152 KB) [file 296_2022_5226_MOESM2_ESM.pdf]

Supplemental Table 1. Exact numbers of longitudinal effect of anti-inflammatory treatment on surrogate markers and disease activity: Intention-to-treat analysis

	IMT (mm)				
	BL	6M	p-value BL vs 6M	48M	p-value BL vs 48M
RA	0.67 (0.02)	0.69 (0.02)	0.257	0.68 (0.02)	0.788
Early RA	0.68 (0.02)	0.68 (0.02)	0.833	0.72 (0.03)	0.160
Established RA	0.67 (0.03)	0.70 (0.03)	0.152	0.64 (0.03)	0.301
Response	0.69 (0.03)	0.69 (0.03)	0.998	0.68 (0.03)	0.698
Non-response	0.65 (0.02)	0.69 (0.03)	0.050	0.65 (0.03)	0.859
	AIx@75 (%)				
	BL	6M	p-value BL vs 6M	48M	p-value BL vs 48M
RA	28 (1)	23 (2)	<0.0001	25 (2)	0.016
Early RA	29 (2)	24 (2)	0.016	24 (2)	0.019
Established RA	27 (2)	22 (2)	0.003	25 (2)	0.359
Response	28 (1)	22 (2)	0.001	25 (1)	0.070
Non-response	28 (3)	25 (3)	0.150	23 (3)	0.124
	PWV (m/s)				
	BL	6M	p-value BL vs 6M	48M	p-value BL vs 48M
RA	8.5 (0.4)	8.0 (0.5)	0.103	8.6 (0.5)	0.900
Early RA	8.7 (0.5)	7.9 (0.6)	0.078	9.1 (0.8)	0.672
Established RA	8.1 (0.6)	8.2 (0.7)	0.832	7.6 (0.6)	0.310
Response	8.6 (0.5)	7.7 (0.4)	0.003	8.4 (0.6)	0.622
Non-response	8.5 (0.6)	8.9 (0.6)	0.552	9.4 (0.9)	0.331
	DAS28-CRP				
	BL	6M	p-value BL vs 6M	48M	p-value BL vs 48M
RA	4.3 (0.1)	2.7 (0.1)	<0.0001	2.4 (0.1)	<0.0001
Early RA	4.4 (0.2)	2.6 (0.2)	<0.0001	2.3 (0.2)	<0.0001
Established RA	4.1 (0.2)	2.8 (0.2)	<0.0001	2.6 (0.2)	<0.0001
Response	4.0 (0.2)	2.0 (0.1)	<0.0001	1.9 (0.1)	<0.0001
Non-response	4.7 (0.2)	3.9 (0.2)	0.001	3.3 (0.2)	<0.0001

Results of linear mixed-effects models with (standard errors), AIx@75: augmentation index normalized to heart rate of 75 beats/minute, BL: baseline, CRP: C-reactive protein, DAS28: disease activity score of 28 joints, IMT: intima media thickness, M: months, PWV: pulse wave velocity, RA: rheumatoid arthritis.

Supplemental Table 2. Exact numbers of longitudinal effect of anti-inflammatory treatment on surrogate markers and disease activity: Per protocol analysis

	IMT (mm)					
	BL	6M	12M	24M	36M	48M
RA	0.68 (0.02)	0.71 (0.02)	0.68 (0.02)	0.69 (0.02)	0.69 (0.02)	0.68 (0.03)
	AIx@75 (%)					
	BL	6M	12M	24M	36M	48M
RA	28 (1)	23 (2)	28 (2)	26 (2)	26 (2)	24 (2)
	PWV (m/s)					
	BL	6M	12M	24M	36M	48M
RA	8.8 (0.4)	8.0 (0.4)	8.3 (0.6)	8.0 (0.4)	8.4 (0.4)	8.5 (0.6)
	DAS28-CRP					
	BL	6M	12M	24M	36M	48M
RA	4.3 (0.1)	2.7 (0.1)	2.5 (0.1)	2.7 (0.2)	2.5 (0.2)	2.3 (0.2)

Results of linear mixed-effects models with (standard errors), AIx@75: augmentation index normalized to heart rate of 75 beats/minute, BL: baseline, CRP: C-reactive protein, DAS28: disease activity score of 28 joints, IMT: intima media thickness, M: months, PWV: pulse wave velocity, RA: rheumatoid arthritis.
