# Supplementary material for: Effect of anti-inflammatory therapy on vascular biomarkers for subclinical cardiovascular disease in rheumatoid arthritis patients
Source: Rheumatol Int. 2022 Oct 21;43(2):315–22. doi: 10.1007/s00296-022-05226-w (PMC9898416; doi:10.1007/s00296-022-05226-w)
Supplement: Supplementary file 3 — Supplementary file3 (PDF 92 KB) [file 296_2022_5226_MOESM3_ESM.pdf]

Supplemental results

Per protocol analysis

In total, 24 (39%) patients were excluded for the per protocol analysis, because of discontinuing treatment of interest during the 48-month period (csDMARDs for early RA or adalimumab for established RA). Reasons for discontinuing treatment were side effects (n=12), treatment failure (n=8), both side effects and treatment failure (n=2), tapering due to good effect (n=1) and practical reasons (n=1). Exact numbers of per protocol analysis of longitudinal effect of anti-inflammatory treatment on surrogate markers and disease activity are shown in Supplemental Table 2.
